# Patients’ perceptions on HIV testing and starting antiretroviral therapy during implementation of universal test and treat

**DOI:** 10.1186/s12889-026-28876-y

**Published:** 2026-08-06

**Authors:** Upasna Singh, Lindani Msimango, Andrew Gibbs, Hlengiwe Shozi, Farzana Osman, Yukteshwar Sookrajh, Paul K. Drain, Nigel Garrett, Jienchi Dorward

**Affiliations:** 1 Centre for the AIDS Programme of Research in South Africa (CAPRISA), University of KwaZulu–Natal, Durban, South Africa; 2 Gender and Health Research Unit, South African Medical Research Council, Pretoria, South Africa; 3 Centre for Rural Health, School of Nursing and Public Health, University of KwaZulu-Natal, Durban, South Africa; 4 Department of Psychology, University of Exeter, Exeter, UK; 5 eThekwini Municipality Health Department, Durban, South Africa; 6 Department of Global Health, Schools of Medicine and Public Health, University of Washington, Seattle, USA; 7 Department of Medicine, School of Medicine, University of Washington, Seattle, USA; 8 Department of Epidemiology, School of Public Health, University of Washington, Seattle, USA; 9 Discipline of Public Health Medicine, School of Nursing and Public Health, University of KwaZulu-Natal, Durban, South Africa; 10 Nuffield Department of Primary Care Health Sciences, University of Oxford, Oxford, UK

**Keywords:** Universal test and treat, HIV, Testing, In-depth interviews

## Abstract

**Introduction:**

Universal Test and Treat (UTT) was introduced to South Africa in 2016 as an HIV prevention and treatment strategy. In this study we aimed to assess the individual perspectives of people living with HIV (PLWH) on starting antiretroviral therapy (ART) during the early implementation of UTT.

**Methods:**

We conducted a thematic analysis of transcripts from interviews with PLWH who had initiated ART and were enrolled in a randomized controlled trial of point-of-care viral load testing in Durban, South Africa.

**Results:**

Twenty-five interviews were conducted between March - August 2018,16 (64%) female, 9 (36%) male PLWH, a median age of 30 years. Asymptomatic participants reported needing time to accept their diagnosis and sought shared decision-making discussions with healthcare-providers about starting treatment. Others appreciated the healthcare-provider leading the decision to treatment, which helped them cope with the shock of testing positive. Many participants indicated being happy with starting treatment early as this meant living a normal life and prevented sickness or death. Some saw UTT as an indication that the public healthcare-system cared for them. For those who tested before UTT implementation, some expressed frustrations about being denied treatment due to high CD4 counts. Policymakers had concerns about stigma being a barrier to UTT and adherence, some participants reported that disclosing their status enabled social support which encouraged adherence.

**Conclusions:**

The implementation of UTT in South Africa has provided a supportive framework for people to understand their diagnosis, accept it and adhere to ART in a caring space.

## Background

Expanding access to antiretroviral treatment (ART) through the introduction of the Universal Test and Treat (UTT) policy has had a tremendous impact on HIV outcomes leading to reductions in morbidity, mortality and infection rates globally [1]. As part of UTT, people who are diagnosed HIV positive, are recommended to start treatment immediately irrespective of their CD4 count [2]. UTT was introduced in 2015 as a treatment and prevention approach following observational and clinical evidence from trials which showed that early use of ART reduces transmission, morbidity, and mortality [3–5]. Prior to the implementation of UTT, people living with HIV (PLWH) in many countries were eligible for ART when their CD4 count was ≤ 500 cells/µl [6]. UTT is critical to strengthening the global response to the HIV pandemic and ensuring that more PLWH access treatment, thereby contributing to the second 95 of the United Nations Program on HIV/AIDS (UNAIDS) 95-95-95 targets which aims at having 95% of all PLWH on treatment [7].

Before the implementation of UTT, public health policy makers were concerned about issues such as adherence, side effects, and drug resistance amongst patients who were newly initiated on ART with high CD4 counts and in the absence of HIV associated morbidity [3]. Other concerns included how feasible it would be to implement UTT in regions where there are a large number of PLWH, such as in sub-Saharan Africa [8], and the potential social impacts of the implementation, such as changes in how people perceive HIV transmission, HIV prevention, testing costs and ART use [8, 9]. The ANRS 12,249 TasP trial emphasized that the community perceived UTT as a tool towards normalization of HIV diagnosis and care [10].

South Africa had an estimated 8.45 million PLWH in 2021, the highest number globally, and has adopted the UTT strategy since September 2016. Cluster randomized trials such as the HPTN 071 Population Effects of Antiretroviral Therapy to Reduce HIV Transmission (PopART) in South Africa and Zambia, and the ANRS 12,249 Antiretroviral Treatment as Prevention (TasP) trials examined the early implementation of UTT at a population level [11, 12]. These trials did not find a clear reduction in HIV incidence with early access to treatment, but did demonstrate some of the facilitators and barriers to reducing HIV incidence during UTT, such as poor linkage to HIV care and the change in the national treatment guidelines, which meant making early ART initiation available in the control arms of the TasP trial [13]. A study conducted by Mshweshwe-Pakela et al. in the Ekurhuleni District in South Africa indicated that same day initiation of ART produced successful outcomes in terms of retention and viral load suppression [14]. Qualitative literature examining barriers and facilitators to UTT found that the major barriers to treatment uptake under UTT included feeling healthy, fear of disclosure, stigma, gender, and concerns about side effects, treatment availability and healthcare worker capacity [15–17]. Facilitators included the motivation to stay healthy and adaptation of services to clients’ specific needs [15]. More qualitative work is required to explore the patients’ perspectives regarding starting treatment under the strategy. In this study, we aimed to assess PLHIV’s perspectives of starting ART during the early implementation of UTT.

## Methods

### Study setting

This qualitative sub-study was embedded within the STREAM trial protocol, with separate informed consent documents for the parent study and qualitative sub-study [18], a clinical trial in which 390 adults living with HIV and on ART were randomized to receive either point-of-care (POC) viral load and CD4 count testing versus standard-of-care laboratory-based viral load testing. The study was undertaken at the Centre for the AIDS Programme of Research in South Africa (CAPRISA) eThekwini Clinical Research Site and the Prince Cyril Zulu Clinic, which provides HIV, tuberculosis, sexual health, and primary care services for people from across the eThekwini region in KwaZulu-Natal, South Africa. Due to the central location of the clinic within a public transport hub, the facility also received clients from areas beyond the eThekwini district. eThekwini accounts for 3.9 million people on ART according to the latest provincial report by the Health Systems Trust [19]. Purposive sampling was used to recruit the 25 participants from across both arms of the parent study for this qualitative sub-study.

### Study design

We conducted semi-structured interviews from March to August 2018 to establish in-depth individual perspectives from clients regarding their opinions and sentiments while starting ART through the UTT programme. Some clients had experienced being tested before UTT was rolled out, and some after UTT was implemented. We used a purposive sampling frame to recruit participants within both arms of the STREAM trial. Eligible participants had exited the STREAM trial and were willing to participate in this qualitative study. Participants were recruited face-to-face or telephonically.

### Data collection

Interviews were conducted at the research clinic using a flexible open-ended topic guide which included participants’ experiences about HIV testing and treatment access. These topic guides were developed through collaboration between the research team and the study nurses and focused on models of HIV care, Central Chronic Medicines Dispensing and Distribution (CCMDD) programme which provides for convenient access to ART for virally suppressed PLWH, and access to care [20, 21]. Interviews were conducted in English or IsiZulu by two trained research assistants. LM (male) was not known to participants. HS (female) had recruited participants into the STREAM trial but was not involved in their clinical care. Audio-recordings were subsequently transcribed and translated into English. We conducted interviews until theoretical saturation was reached and no new concepts were identified. Interviews lasted 30–60 min.

### Data analysis

After data collection had finished, we (LM, US, JD, AG) performed data coding and analysis using NVivo 12 software (QSR International, Melbourne, Australia), employing a thematic content analysis. To do this, LM, US and JD developed a codebook which identified codes, definitions, and example quotes. We used inductive coding to understand participant’s perspectives regarding HIV testing and treatment access during UTT.

Transcription and translation of the interviews were conducted prior to the coding process. These were carried out independently by LIM, an experienced team member fluent in IsiZulu and English. Each transcript was transcribed verbatim and was subsequently translated into English and a quality check was performed by another team member to confirm consistency and correctness.

Coding and analysis were predominantly conducted by LM, supported by US, JD and a senior qualitative researcher (AG) and there was continual discussion among the team about the themes and sub-themes as coding occurred. Participants did not review the analysis. All twenty-five transcripts were reviewed. The data was then placed into themes according to the codebook.

### Ethics

The study received ethical approval from the University of KwaZulu-Natal Biomedical Research Ethics Committee (BE648/17) and the University of Washington Institutional Review Board (STUDY00001466). Written informed consent was obtained from all participants for the main study and separately for the qualitative sub-study. To ensure anonymity and confidentiality, participant’s identifying information was not transcribed and any names and identifying descriptions mentioned in the audio recordings were given fictitious names and descriptions.

## Results

Purposive sampling frame was used to recruit participants within both arms of the STREAM trial, and eligible participants had exited the STREAM trial and were willing to participate in this qualitative study. We conducted interviews, between March and August 2018,with 25 participants who had reached their 18 month post-ART initiation timepoint. Of this group, 16 (64%) were female and 9 were(36%) male, of median age 30 years (interquartile range [IQR] 27–37). Their baseline median CD4 counts were 264 cells/mm^3^(IQR 171–503) (Table 1).

**Table 1:** Demographics of the twenty-five participants who were interviewed

		Frequency (*N*=25)	%
Gender	Male	9	36.00
	Female	16	64.00
Initiation CD4 count	≤ 200	9	36.00
	201–350	6	24.00
	351–500	2	8.00
	> 500	8	32.00
Baseline CD4 count	Median (IQR)	264 (171–503)	
Age at time of HIV diagnosis	Median (IQR)	30 (27–37)	
Participants who tested HIV-positive prior to UTT		28% (7/25)	
Participants who tested HIV-positive after UTT		72% (18/25)	
Time from diagnosis to initiation on ART	≤ 6 months	16	66.67
	> 6 months	8	33.33

The themes that emerged from the analysis included reactions to testing HIV positive during UTT, their opinions of pre-initiation counselling, how UTT access helped people accept their diagnoses, their help seeking trends when faced with side effects of ART, adherence, and disclosure to their partners or immediate social circle. We further elaborate on these themes below.

### Reactions to testing positive during UTT and counselling about starting treatment

Participants expressed varied reactions to their HIV positive diagnosis. Some participants were shocked and took time to come to terms with accepting their status. However, through extensive counselling, they were able to accept their diagnosis.

*“I was shocked… everybody gets shocked*,* but I told myself*,* it’s not a problem because people live*,* there are people from where I live who are like this*,* but I’ve never heard that they are dying and at home my cousin has it and is living with no problems. So*,* I accepted it when they told me. I went on and they told me that I’m HIV positive and I accepted it*,* I went for counselling and was told it’s not a death sentence*,* you will get medication.” Female*,* 40–50 years*

Some participants described a degree of shame and attempted to hide their status from their family members.

*“I went for counselling and was told it’s not a death sentence*,* you will get medication*,* I went home and I never told anyone*.” *Male*,* 20–30 years*

Despite feeling emotionally distressed, some participants understood that this was an opportunity for them to access treatment early and agreed to immediately initiate ART based on the understanding that this would help them live a normal, healthy life.

*“To be honest it was difficult*,* it wasn’t easy to accept that I’m positive*,* it was hard so much that even at home I struggled to sleep after knowing my status…it broke me a lot …you just need to stand up for your life and take action” Male*,* 20–30 years*

*“It was very difficult I do not want to lie*,*…I was a virgin*,* I didn’t get HIV through sex…the lady helped me to accept my situation*,* it was difficult to accept but then at the end I ended up accepting and then I was fine”. Female*,* 20–30 years*

Participants also stated that the change in their HIV status did not have any impact on their identity as an individual. The immediate availability and option to initiate ART contributed to them being willing and able to start treatment immediately with little obvious ‘struggle’.

*“I didn’t feel like a different person*,* I didn’t see any difference*,* I saw myself as a normal person who needs to take care of herself and take her medication properly so life can go on. I didn’t feel any change and I wasn’t sick at all.” Female*,* 30–40 years*

*“So*,* we heard from a friend with my roommate whom I stayed with at res [university halls of residence] that there is research going on here. So*,* you just come and test and do everything and then they give you something. Ok so we decided that we will come and test because I have never tested before. So*,* I came*,* and we tested and that is when I found my results and I started taking medication.” Female 20–30 years*

### The role of universal treatment access in helping people accept their diagnosis and starting treatment

UTT helped people to accept their diagnosis and willingly start treatment. Among those who started treatment immediately they described how they appreciated having an open discussion with their healthcare providers regarding starting treatment and being provided with a choice to be initiated on ART. This open discussion encouraged them to make the decision of starting ART immediately.

*“They took me to another sister who tested me and then took bloods and told me when to come back to check for my CD4 count results. When I got back*,* they saw it was high and they told me about test and treat program and told me it’s up to me to decide whether I want to start with the medication or wait until it drops. I said I want to start”*. *Female*,* 20–30 years*

*“In 2003 I think the medication was not available. I then gave birth…oh they gave a pill to prevent the child. I prevented it*,* in 2013 i think*,* I kept on visiting the clinic and then I started the medication”. Female*,* 20–30 years*

For a few participants, the decision to start treatment was led by education from the healthcare provider. Some described that their understanding of HIV, through UTT healthcare providers, persuaded them to start ART:

*“Yes*,* they said I will start taking my medication*,* oh I took the bloods and they said you don’t wait for the CD4 count*,* mine was 537*,* they said you don’t wait anymore because your CD4 count is high. You don’t wait anymore you can start taking your medication. I said it’s okay*,* I can take them as long as there won’t be any problems*,* and they tell you that they will have side-effects at the beginning*,* I took them and they did have some side-effects they told me I will have bad dreams*,* I did have a bad dream on the first day.” Female*,* 20–30 years*

Participants found emotional strength and solace in being able to start ART, and in doing so take control of a small aspect of their life, which helped them cope with the shock of testing HIV positive and maintaining privacy surrounding their status after starting ART.

*“To be honest it was difficult*,* it wasn’t easy to accept that I’m positive*,* it was hard so much that even at home I struggled to sleep after knowing my status. When I got the results that my CD4 count was 300 and something*,* it broke me a lot. But I told myself that this can happen to anyone you just need to stand up for your life and take action. That is why at home no one knows up until now. I’m not saying I’m proud of that but it’s because most of the time I’m alone here”. Male*,* 20–30 years*

Although many people were keen to start treatment immediately and saw it as an important way of taking control of their diagnosis, others required more time to come to terms with it and chose to start at a later date.

*“They told me to come back in September and I came to take bloods and they said if I want to start taking medication*,* I can start. I didn’t start them*,* I wasn’t ready.” - Female 20–30 years*

### Benefits of UTT and how people navigated the potential downsides

Most participants who started treatment because of UTT rather than getting sick or having a low CD4 count emphasized that being initiated on treatment early yielded several benefits. Among the benefits highlighted by participants was that early access to treatment prevented them from developing serious illness or dying.

*“You see this test and treat thing of yours*,* had you done it earlier*,* people would not have died this much from HIV. Because this test and treat thing is working. If it was for me*,* the whole world would have done this from the beginning*,* it has a great impact.” Female 40–50 years*

Participants were provided with detailed education on the use of ART, the effect of the treatment on HIV viral load and CD4 counts, and they were alerted to the possible side effects of each component of their treatment. Advice was provided to them regarding how to prevent or manage the common side effects, and they were told to seek medical attention and advice if they felt ill whilst on treatment.

*“Oh*,* it happened when I was … at home after I started medication*,* I was not well*,* I had stomachache*,* I went to the nearby hospital*,* and I told them that I have a stomachache and told her that I just started medication. The nurse who helped me told me that sometimes when you start the medication*,* there will be issues but when time goes by it gets fine. But she said if the problem persists*,* I should come back at the hospital.”* Female 20–30 years

Participants recognize that UTT helped to prevent illness and death, and this was seen by some as being evident that the public healthcare system cares for them.

“*I saw that they know their job*,* they don’t wait for you to get sick and then initiate treatment*,* because even if virus is small*,* they give you treatment and you start at that time. I saw that they are people who care for other people. I was very happy*.”- *Female 20–30 years*

Participants took note of the people around them who were also accessing ART and this assisted them in realizing that the treatment would allow them to live normal, healthy lives.

*“I would see people and they look healthy when we are sitting outside and when we get upstairs a person is following me. Others would come because they are sent by someone but you would hear others talking and I would realize that I am not alone and I shouldn’t stress about this.” Male*,* 30–40 years*

Despite the previous concerns from policymakers that HIV related stigma might cause poor adherence to ART and thereby hinder UTT, some participants highlighted that disclosing their statuses enabled social support and encouraged them to take their treatment.

*She [the participant’s sister] would encourage me a lot*,* everyone at home as I’ve said*,* when I’m this side my aunt encourages me*,* when I go to [location mentioned] my mother and father are good people there is no one I would say doesn’t know my status I would be lying*,* even my child knows.” Female*,* 20–30 years*

*“I’m a person who’s not secretive talk*,* so I won’t have a problem even if the house is full and it is 8’o’clock I have to take my medication. The people I stay with I tell them that I’m a person who is concerned about her health” Female*,* 30–40 years*

## Discussion

### Summary of findings

This study assessed patients’ perspectives on starting ART during the early implementation of UTT. We found that UTT played a role in helping newly diagnosed participants to accept their HIV positive status. Three aspects were central to this. First, the sense that UTT has changed the way that some clients feel perceived by the healthcare system, with them now seeing early ART initiation as evidence of respect and care. Second, was the role of the healthcare provider and the space that was provided in to discuss their diagnosis and the potential benefits of UTT. Third, being able to start ART early, rather than waiting for their CD4 count to drop, provided people with a strategy to exert some self-efficacy and control after being diagnosed with HIV. Additionally, UTT provided the benefit of preventing participants from developing opportunistic or AIDS related illnesses prior to ART initiation. The increasing number of people on ART in the community after UTT was implemented created an avenue for increased access to pee support.

### Relation to literature

Much of the qualitative literature around UTT comes from five different UTT trials in different parts of Sub-Saharan Africa [22], with four out of five of these trials examining social aspects and patient perceptions of UTT.

A significant barrier to accessing HIV testing and ART has been the mistreatment people experience at the hands of healthcare professionals [23]. For clients who saw the provision of medicine and counselling through UTT as an aspect of care, this care then enabled them to accept their new diagnosis of living with HIV. An ethnographic study of the POPART trial examined community care-givers who were responsible for enacting various steps in the care cascade (HIV testing, linking people who tested positive to care, and supporting adherence), and found that enactment of UTT involved much broader forms of care-giving, including emotional and social support beyond merely providing ART to clients [24], reflecting our findings that UTT can represent a sense of wider care from the health system.

Regarding the role of counselling and messaging around UTT, a study of the MaxART trial in eSwatini found that framing UTT as access to earlier treatment, and taking responsibility for one’s own health, rather than focusing on treatment as prevention, was acceptable [25]. Another qualitative study from eSwatini found that starting ART as part of UTT was seen as important for maintaining vitality and productivity, particularly among men [26]. However, some participants in this study also expressed doubts about the benefits of ART given that they were asymptomatic at the time of HIV diagnosis. Early studies investigating the efficacy of UTT mentioned concerns regarding adherence, disclosure and treatment side-effects [3], in our study some patients highlighted the benefit of starting treatment early was that it would prevent them from dying or getting sick. This reflects findings from the START and TEMPRANO trials [4, 5]. Our findings were similar to those found in a study by Scott Batey et al. where patient perceptions of acute care HIV testing in an Emergency Room setting were investigated [15, 27]. Batey et al. found that 85.8% of patients supported the concept of testing and receiving treatment for HIV as a routine approach to care, emphasizing that this was an opportunity for knowledge sharing, prevention, convenience, and treatment.

In rural KwaZulu-Natal, South Africa, a study examining barriers and facilitators to universal ART uptake found that several factors contributed to reduced uptake, the most prevalent reason in this population being stigma [13]. Most participants in our study did not report stigma as a barrier to UTT, but we did not have any participants who had not started ART. Our findings are similar to that of Scott N.A. et al., where patient perceptions of UTT included those of both urban and rural settings and the qualitative data supported the theme of UTT offering a caring, non-judgmental, easily accessible space for people wanting to test for HIV and those living with HIV [28].

The social, logistical and individual barriers to accessing HIV testing and treatment in the absence of UTT are brought to the fore through interviews conducted in a study by Earnshaw et al. in Gugulethu Township, Western Cape, South Africa [29]. More recent studies have investigated retention in care following the implementation of UTT and emphasize the effect of these barriers on retention [2, 17]. In addition to logistical and system barriers, there are also patient-specific barriers which include food insecurity, substance abuse, finances for travel to the clinics and perceived stigma [15, 30].

### Strengths and limitations

Most of our participants were diagnosed shortly after UTT was implemented in South Africa and were enrolled in the STREAM study when they had been on treatment for more than 6 months. Therefore, they were able to reflect on the issues around accessing ART during the early implementation of UTT. The interviews were held as open discussions rather than structured questionnaires, which allowed the participant to feel more comfortable in answering questions honestly and without any bias. The interviewers were not involved in the testing and initiation process, thus providing an element of confidentiality to the interviews, thereby making the participants feel comfortable talking freely.

The Ethekwini municipality, within the province of KwaZulu-Natal, is known to be a high burden region for HIV. The data used in the study was extracted from 25 patient’s interviews which may have not been representative of the study population or the wider public, and so limits the generalizability of our findings to other contexts. Similarly, the views obtained are those of people who were initiated on ART only and have not taken into consideration the opinions of those who are ART naïve. The main objective of the study was to assess point-of-care viral load testing and differentiated HIV care [17, 22], with perspectives on UTT evaluated here as a sub-study.

### Implications for research and policy

Our research supports the rollout of UTT and has implications for how UTT strategies could be improved. Firstly, ensuring that ART provision is provided in a client centered manner that reinforces the sense of UTT as part of a caring healthcare system may help to increase ART uptake and retention in care. Second, our findings highlight the importance of comprehensive counselling from healthcare workers around the time of ART initiation, that takes into account clients varied responses to being HIV positive and is tailored to provide support and meet their individual needs. Third, as UTT means the numbers of people receiving ART in the community increases, our findings highlight the potential for increased use of peer support to help people navigate initiating and staying on ART. Further research is needed about populations who test positive and refuse ART initiation, who were not included in our study. The positive feedback from participants indicates the need to investigate how UTT can be sustained and offered to the wider population in urban, peri-urban and rural settings throughout Sub-Saharan Africa.

## Conclusions

The implementation of UTT in South Africa may provide a supportive framework for people to have access to ART, make sense of their diagnosis and provide a caring space within the context of this study setting. However, further work is required to maximize the benefits of UTT.

## Data Availability

The datasets used and/or analyzed during the current study can be requested by any bona fide researcher from the corresponding author or through the CAPRISA website: https://www.caprisa.org/Pages/CAPRISAStudies.

## References

[1] Cohen MS, Chen YQ, McCauley M, Gamble T, Hosseinipour MC, Kumarasamy N, et al. Antiretroviral therapy for the prevention of HIV-1 transmission. N Engl J Med. 2016;375(9):830–9. 10.1056/NEJMoa1600693 PMC5049503 27424812

[2] Hirasen K, Fox MP, Hendrickson CJ, Sineke T, Onoya D. HIV treatment outcomes among patients initiated on antiretroviral therapy pre and post-universal test and treat guidelines in South Africa. Ther Clin Risk Manag. 2020:169–80. 10.2147/TCRM.S227290 PMC7061415 32184609

[3] Geffen N, Low MO. When to start antiretroviral treatment? A history and analysis of a scientific controversy. South Afr J HIV Med. 2017;18(1):8. 10.4102/sajhivmed.v18i1.734 PMC11500577 39450053

[4] Katz IT, Siedner MJ. Does early ART in sub-Saharan Africa decrease mortality? Lancet HIV. 2015;2(9):e354–5. 10.1016/S2352-3018(15)00130-7 PMC4634669 26423540

[5] Group TAS. A trial of early antiretrovirals and isoniazid preventive therapy in Africa. N Engl J Med. 2015;373(9):808–22. 10.1056/NEJMoa1507198 26193126

[6] Bor J, Ahmed S, Fox MP, Rosen S, Meyer-Rath G, Katz IT, et al. Effect of eliminating CD4-count thresholds on HIV treatment initiation in South Africa: An empirical modeling study. PLoS ONE. 2017;12(6):e0178249. 10.1371/journal.pone.0178249 PMC5472329 28617805

[7] Heath K, Levi J, Hill A. The Joint United Nations Programme on HIV/AIDS 95-95-95 targets: worldwide clinical and cost benefits of generic manufacture. Aids. 2021;35(Supplement 2):S197–203. 10.1097/QAD.0000000000002983 34115649

[8] Reynolds LJ, Camlin CS, Ware NC, Seeley J. Exploring critical questions for the implementation of universal test and treat approaches to HIV prevention and care. Taylor & Francis; 2016. pp. 1–6. 10.1080/09540121.2016.1178960 27421046

[9] Mukora R, Smith HJ, Herce ME, Chimoyi L, Hausler H, Fielding KL, et al. Costs of implementing universal test and treat in three correctional facilities in South Africa and Zambia. PLoS ONE. 2022;17(8):e0272595. 10.1371/journal.pone.0272595 PMC9409581 36006967

[10] Orne-Gliemann J, Larmarange J, Boyer S, Iwuji C, McGrath N, Bärnighausen T, et al. Addressing social issues in a universal HIV test and treat intervention trial (ANRS 12249 TasP) in South Africa: methods for appraisal. BMC Public Health. 2015;15(1):1–14. 10.1186/s12889-015-1344-y PMC4351958 25880823

[11] Iwuji C, Chimukuche RS, Zuma T, Plazy M, Larmarange J, Orne-Gliemann J, Seeley J. Test but not treat: Community members’ experiences with barriers and facilitators to universal antiretroviral therapy uptake in rural KwaZulu-Natal, South Africa. PLoS One. 2020;15(9):e0239513. 10.1371/journal.pone.0239513 PMC7514038 32970730

[12] Thomas R, Probert WJ, Sauter R, Mwenge L, Singh S, Kanema S, et al. Cost and cost-effectiveness of a universal HIV testing and treatment intervention in Zambia and South Africa: evidence and projections from the HPTN 071 (PopART) trial. Lancet Global Health. 2021;9(5):e668–80. 10.1016/S2214-109X(21)00034-6 PMC8050197 33721566

[13] Brault MA, Spiegelman D, Abdool Karim SS, Vermund SH. Integrating and interpreting findings from the latest treatment as prevention trials. Curr HIV/AIDS Rep. 2020;17:249–58. 10.1007/s11904-020-00492-4 PMC7260110 32297219

[14] Mshweshwe-Pakela N, Hansoti B, Mabuto T, Kerrigan D, Kubeka G, Hahn E, Hoffmann CJ. Feasibility of implementing same-day antiretroviral therapy initiation during routine care in Ekurhuleni District, South Africa: Retention and viral load suppression. South Afr J HIV Med. 2020;21(1):1–6. 10.4102/sajhivmed.v21i1.1085 PMC7479383 32934830

[15] Iwuji C, Chimukuche RS, Zuma T, Plazy M, Larmarange J, Orne-Gliemann J, et al. Test but not treat: Community members’ experiences with barriers and facilitators to universal antiretroviral therapy uptake in rural KwaZulu-Natal, South Africa. PLoS ONE. 2020;15(9):e0239513. 10.1371/journal.pone.0239513 PMC7514038 32970730

[16] Johannessen A. Are men the losers of the antiretroviral treatment scale-up? Aids. 2011;25(9):1225–6. 10.1097/QAD.0b013e32834403b8 21593620

[17] Nicol E, Mehlomakulu V, Jama NA, Hlongwa M, Basera W, Pass D, et al. Healthcare provider perceptions on the implementation of the universal test-and-treat policy in South Africa: a qualitative inquiry. BMC Health Serv Res. 2023;23(1):293. 10.1186/s12913-023-09281-2 PMC10045036 36978086

[18] Dorward J, Garrett N, Quame-Amaglo J, Samsunder N, Ngobese H, Ngomane N, et al. Protocol for a randomised controlled implementation trial of point-of-care viral load testing and task shifting: the Simplifying HIV TREAtment and Monitoring (STREAM) study. BMJ open. 2017;7(9):e017507. 10.1136/bmjopen-2017-017507 PMC5623564 28963304

[19] Ndlovu N, Padarath A, editors. District Health Barometer 2022/23. Durban: Health Systems Trust; 2024.

[20] Dorward J, Msimango L, Gibbs A, Shozi H, Tonkin-Crine S, Hayward G, et al. Understanding how community antiretroviral delivery influences engagement in HIV care: a qualitative assessment of the Centralised Chronic Medication Dispensing and Distribution programme in South Africa. BMJ open. 2020;10(5):e035412. 10.1136/bmjopen-2019-035412 PMC7245406 32414827

[21] Liu L, Christie S, Munsamy M, Roberts P, Pillay M, Shenoi SV, et al. Expansion of a national differentiated service delivery model to support people living with HIV and other chronic conditions in South Africa: a descriptive analysis. BMC Health Serv Res. 2021;21:1–8. 10.1186/s12913-021-06450-z PMC8127180 34001123

[22] Perriat D, Balzer L, Hayes R, Lockman S, Walsh F, Ayles H, et al. Comparative assessment of five trials of universal HIV testing and treatment in sub-Saharan Africa. J Int AIDS Soc. 2018;21(1):e25048. 10.1002/jia2.25048 PMC5810333 29314658

[23] Threats M, Boyd DT, Diaz JE, Adebayo OW. Deterrents and motivators of HIV testing among young Black men who have sex with men in North Carolina. AIDS Care. 2021;33(7):943–51. 10.1080/09540121.2020.1852161 PMC8155091 33244990

[24] Wademan DT, Reynolds LJ. Interrogating concepts of care in the HIV care continuum: ethnographic insights from the implementation of a Universal Test and Treat approach in South Africa. AIDS Care. 2016;28(Suppl 3):52–8. 10.1080/09540121.2016.1161164 26984394

[25] Vernooij E, Mehlo M, Hardon A, Reis R. Access for all: contextualising HIV treatment as prevention in Swaziland. AIDS Care. 2016;28(Suppl 3sup3):7–13. 10.1080/09540121.2016.1178954 27421047

[26] Horter S, Wringe A, Thabede Z, Dlamini V, Kerschberger B, Pasipamire M, et al. Is it making any difference? A qualitative study examining the treatment-taking experiences of asymptomatic people living with HIV in the context of Treat-all in Eswatini. J Int AIDS Soc. 2019;22(1):e25220. 10.1002/jia2.25220 PMC6351702 30697970

[27] Scott Batey D, Ryan Cantor VLH, Hamlin CM, Ross-Davis K, Nevin C, Zimmerman C, Thomas S, Mugavero MJ. Willig. Short Communication Routine HIV Testing in the Emergency Department: Assessment of Patient Perceptions. AIDS Res Hum Retroviruses. 2012;28(4):352–6. 10.1089/aid.2011.0074 PMC3316117 21790474

[28] Scott NA, Maskew M, Fong RM, Olson IE, Brennan AT, Fox MP, et al. Patient Perspectives of Quality of the Same-Day Antiretroviral Therapy Initiation Process in Gauteng Province, South Africa: Qualitative Dominant Mixed-Methods Analysis of the SLATE II Trial. The Patient -. Patient-Centered Outcomes Res. 2021;14(2):175–86. 10.1007/s40271-020-00437-4 PMC7884580 32909218

[29] Earnshaw VA, Bogart LM, Courtney I, Zanoni H, Bangsberg DR, Orrell C, et al. Exploring treatment needs and expectations for people living with HIV in South Africa: a qualitative study. AIDS Behav. 2018;22:2543–52. 10.1007/s10461-018-2101-x PMC6051887 29619585

[30] Nicol E, Jama NA, Mehlomakulu V, Hlongwa M, Pass D, Basera W, et al. Enhancing linkage to HIV care in the Universal Test and Treat era: Barriers and enablers to HIV care among adults in a high HIV burdened district in KwaZulu-Natal, South Africa. BMC Public Health. 2023;23(1):1756. 10.1186/s12889-023-16576-w PMC10492313 37689667

